# The DTX Protein Family: *An Emerging Set of E3 Ubiquitin Ligases in Cancer*

**DOI:** 10.3390/cells12131680

**Published:** 2023-06-21

**Authors:** Pierluigi Scalia, Stephen J. Williams, Antonio Suma, Vincenzo Carnevale

**Affiliations:** 1 ISOPROG-Somatolink EPFP Research Network, Philadelphia, PA 19102, USA; 93100 Caltanissetta, Italy; 2 Sbarro Institute for Cancer Research and Molecular Medicine, Temple University, Philadelphia, PA 19122, USA; 3 Institute of Computational Molecular Science, College of Science and Technology, Temple University, Philadelphia, PA 19122, USA

**Keywords:** Deltex, DTX, RNF, Ring Finger (domain), RING, really interesting new gene (motifs), H2-RING, synonym of C3H2C3-RING, HC-RING, synonym of C3HC4-RING, DTC, Deltex C-terminal (region), UbE3L, Ubiquitin E3 Ligase, Degron, *p*Degron, Phospho-Degron, *pi*Degron, Phospho-inhibited Degron

## Abstract

Until recently, Deltex (DTX) proteins have been considered putative E3 ligases, based on the presence of an E3 RING domain in their protein coding sequence. The human DTX family includes DTX1, DTX2, DTX3, DTX3L and DTX4. Despite the fact that our knowledge of this class of E3-ubiquitin ligases is still at an early stage, our understanding of their role in oncogenesis is beginning to unfold. In fact, recently published studies allow us to define specific biological scenarios and further consolidate evidence-based working hypotheses. According to the current evidence, all DTX family members are involved in the regulation of Notch signaling, suggesting a phylogenetically conserved role in the regulation of this pathway. Indeed, additional evidence reveals a wider involvement of these proteins in other signaling complexes and cancer-promoting mechanisms beyond NOTCH signaling. DTX3, in particular, had been known to express two isoform variants (DTX3a and DTX3b). The recent identification and cloning of a third isoform variant in cancer (DTX3c), and its specific involvement in EphB4 degradation in cancer cells, sheds further light on this group of proteins and their specific role in cancer. Herein, we review the cumulative knowledge of this family of E3 Ubiquitin ligases with a specific focus on the potential oncogenic role of DTX isoforms in light of the rapidly expanding findings regarding this protein family’s cellular targets and regulated signaling pathways. Furthermore, using a comparative and bioinformatic approach, we here disclose a new putative motif of a member of this family which may help in understanding the biological and contextual differences between the members of these proteins.

## 1. Introduction

RING (Really Interesting New Gene) Fingers (RNF) domain-containing proteins constitute the most widely represented type of E3 ubiquitin ligases in the human genome. They are involved both in the regulation of targeted-proteins’ function as well as in ubiquitin proteasome system (UPS)-mediated degradation (reviewed by [1]). RING finger domains found in RNF proteins bind two zinc ions in a “cross-brace” fashion involving a distinctive cysteine and histidine residue-containing motif, a feature previously known to be shared only by zinc-finger DNA-binding proteins [2]. Despite some DTX proteins having been found overexpressed in cancer, their mechanistic involvement in the underlying pathological process is still at an early stage of investigation [3]. Most ubiquitin E3 ligases (UbE3L) studied until recently had been found to be involved in the N-degradation pathway responsible for recruiting specific UbE3L to the targeted proteins’ N-terminal region [4]. Recently, a growing number of C-Degrons-bearing proteins have been identified, along with the underlying UbE3L-driven degradation complexes (reviewed by Varshawsky [5]). The relatively recent field of C-Degron pathways had lead some authors to define this type of degradation mechanism with the acronym of “DesCEnd” (from “Destruction by the C-End”) [6]. Although to date the RING motifs in DTX proteins have not been restricted to either of the N-Degron or C-Degron degradation pathways, the recently described involvement of DTX3c in the specific recruitment to the EphB4 C-*pi* (phospho-inhibited) Degron [7,8] assigns DTX3 to the C-Degron pathway. The present review focuses on what we have learned so far about the human DTX class of the UbE3L family, with a specific focus on their role, cellular targets and regulated signaling pathways in cancer, and in light of the lessons learned through the recent literature and the above-mentioned newly identified cancer-specific variant. 

## 2. DTX Proteins Structural and Functional Features: Phylogenetic and Intra-Species Considerations

**Figure 1:**
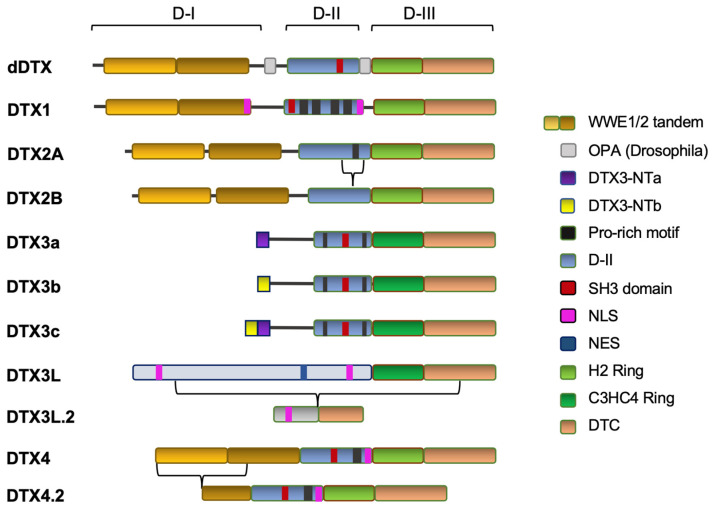
The *Drosophila* and human DTX protein domain structure. WWE, WWE tandem domains; DTX3-NTa, DTX3a N-Terminal variant (isoform 1); DTX3-NTb, DTX3b N-Terminal variant (isoform 2), D, D-Domain; H2-RING, canonical Ring finger motif (H2)-containing domain; HC-RING, C3HC4 finger motif-containing domain; DTC, Deltex C-Terminal Domain. DTX3c N-Terminal domain retains both 1a and 1b exons to generate a new isoform (isoform 3) with N-terminus 3D unique distribution differing from the canonical DTX3a and DTX3b isoforms.

DTX protein expression is almost ubiquitous in mammalians, although they display the highest expression in specific cell and tissue types like blood vessels, embryonic neural tissues, genital and reproductive apparatus, pancreas, lungs, liver, kidneys, adrenal gland, skin, intestinal epithelial components and muscle (see Uberon Anatomy Ontology at https://bioportal.bioontology.org/ontologies/UBERON accessed on 17 May 2023 [9]). A first hint at the physiological role of DTX proteins has come from a DTX *Drosophila* gene null mutant study which confirmed the tissue-specific requirement for DTX expression and its dispensable role in embryo development [10]. This is consistent with the finding that T-cells, a DTX1 and 2 expressing mammalian cell type, can develop normally in absence of both DTX1 and DTX2 [11]. On the other hand, overexpression of DTX proteins in mammalian systems has been found to affect myogenesis, neurogenesis [12] and the proper function of lymphoid organs [13]. More recently, DTX protein expression in cancer cells has revealed a wide landscape of contextual functions linked, in part, to their regulation of NOTCH signaling. As graphically summarized in Figure 1, mammalian DTX proteins share all or part of the *Drosophila* DTX (dDTX) progenitor structure [12,14]. As such, some phylogenetic and intra-species key comparative points can be made per each *Drosophila* reference domain structure in order to better understand the emerging cellular and systemic functions of DTX proteins in mammalians. In particular, human DTX proteins are linked to the expression of five paralog genes along with their main isoform variants (Table 1 and Figure 1). A number of human isoform variants have been reported for DTX proteins (Figure 1). These are discussed below in relationship with the underlying protein domain features.

### 2.1. DTX Domain I: WWE Domain-Bearing and WWE-Domain-Less DTX Proteins

The domain structure of human DTX proteins reflects that observed in *Drosophila* DTX which share a phylogenetically conserved three-domains structural organization. Starting from the N-terminal region, *Drosophila* DTX bears a tandem WWE domain which is conserved in DTX1, −2, and −4 human paralogs (Figure 1). WWE tandem domain in dDTX was shown to bind NOTCH ankyrin repeats [15], and its presence in human DTX1, DTX2 and DTX4 N-terminal domain supports their ability to physically interact with NOTCH ankyrin motifs [14]. This finding further strengthens the functional link between DTX proteins and Notch signaling regulation. Interestingly, WWE tandem domains are frequently found in ubiquitin UbE3Ls and ADP-ribose polymerases [16] although the direct implication of such occurrence has not yet found a mechanistic explanation. In light of this finding, it is not surprising that some WWE-domain bearing DTX members such as DTX2 have been found associated to the recruitment and regulation of ADP-ribosylation targets [17,18].

### 2.2. Domain II: The Proline-Rich Region in DTX Proteins

The N-terminal WWE tandem domain in dDTX, DTX1, DTX2 and DTX4 (part of the interspecies domain-I), is followed by a proline-rich region which stands as the constitutive component of DTX domain-II. This proline-rich region is present also in DTX3, following a short unique N-terminus domain. The only DTX member missing this proline-rich region is DTX3L, which displays a two-domain structural organization (Figure 1) with a long and unique N-terminus domain. DTX3L N-terminus domain mediates homo- and hetero-dimeric binding, and it enhances its heterodimeric self-ubiquitination when bound to DTX1 in vitro [14]. A dDTX study found DTX proline-rich region (domain-II) to contribute to NOTCH regulation since a deletion mutant spanning the full dDTX proline-rich domain (aa 476–484) exhibited a dominant negative effect on Notch activation [19]. Another hint on the function of DTX domain-II has come from the identification of an SH3 motif in dDTX (aa 480–484) along with its ability to bind the signaling adaptor GRB2 [20]. Interestingly, the SH3 motif is conserved in DTX1, DTX3 and DTX4 but is not present in human DTX2 proline-rich domain (see Figure 1), offering the first structural hint to DTX2’s physical interaction differences underlying its diversified cellular functions. Indeed, a hint of this SH3 motif potential functions in DTX2 proline-rich domain may come from the study of its isoform (DTX2B), which lacks this SH3 motif. Specifically, DTX2B displays the exclusion of DTX2A fourth exon (aa 337–383), which contains a serine residue (S360) which has been found phosphorylated by cell-cycle-related kinases (Aurora and polo-like kinases) during mitosis by a large-scale phospho-proteomic study [21]. Therefore, this DTX2 residue embedded in its proline-rich domain could, *per se*, offer a first hint at DTX2’s potential isoform-specific functional consequences with regard to proliferative disfunctions such as in cancer.

### 2.3. Domain III: The Deltex C-Terminal Domain (DTC) and the H2- and HC-RING Domains in DTX Family Members 

**Figure 2:**
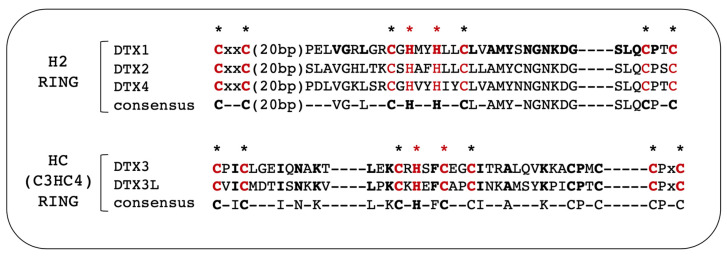
Sequence comparison and consensus (bold) of the RING motifs in DTX proteins. Bolded sequences, consensus sequence within each RING category, red letters under the asterisk indicate consensus residues shared among all DTX proteins RING motifs; black asterisks are the conserved C residues concurring to the Ring domain numbering; red asterisks correspond to positions in the RING domain alignment supporting the H2 versus HC RING acronym.

A common feature shared by all DTX proteins relates to the presence of a RING domain in their DTC region [22]. Although the UbE3L enzymatic activity has not been yet demonstrated for all RING-motifs containing proteins, the putative UbE3L enzymatic nature of DTX proteins has received sufficient experimental support in that all of them have been shown to (a) bear self-ubiquitination capability as homodimer or heterodimers [19,23]; (b) bind and increase the ubiquitination of at least one cellular target either in cell-free or in vivo cellular assays (Table 2); and (c) interact with an E2-ubiquitin conjugase (URL source: UNIPROTKB -> BIOGRID [https://www.uniprot.org/database/DB-0184; Release 2023_02 accessed 7 May 2023 [24]). Since two types of RING motifs are found in DTX proteins, the RING-type motif present in DTX ligases can be used to further sub-categorize them. Specifically, the H2-RING motif is found in DTX1, DTX2 and DTX4, while an HC-RING (synonym of C3HC4-RING) domain is present in DTX3 and DTX3L (Figure 1 and Figure 2). The name of the H2-RING refers to the histidine residues present in the “HxxH” core motif spanning the central part of the RING domain as shown in Figure 2 [22]. Similarly, the C3HC4-RING motif featured in DTX3 and DTX3L at the same conserved core location, bears an “HxxC” motif from which originates its acronym of HC-RING. The biochemical and biological significance associated to these two types of RING motifs in the context of DTX proteins biological function is still unclear. However, the finding that the HC-RING of DTX3L is able to enhance self-ubiquitination of the DTX3L when dimerizing with DTX1 containing an H2-RING in vitro [14], suggests that HC-RING and H2-RING DTX heterodimers may also play a synergistic role in vivo. Additionally, these two RING motifs functions are likely to be affected by intra-molecular interaction with other DTX domains ultimately conferring them specificity for their cellular binding partners and substrates. Interestingly, the DTX RING bears also non enzymatic functions as suggested by the mutagenesis of aa 571 located within the *Drosophila* DTX RING-H2 (spanning aa 548–603) which impairs dDTX multimerization when associated to residue 574 (->A) replacement [19]. This finding suggests a cooperative function of specific residues within the RING structure towards modulating DTX homologous and heterologous configuration. 

### 2.4. NLS and NES Motifs Present in DTX Proteins: The Basis for Cellular Localization and Function

#### 2.4.1. DTX1

**Figure 3:**
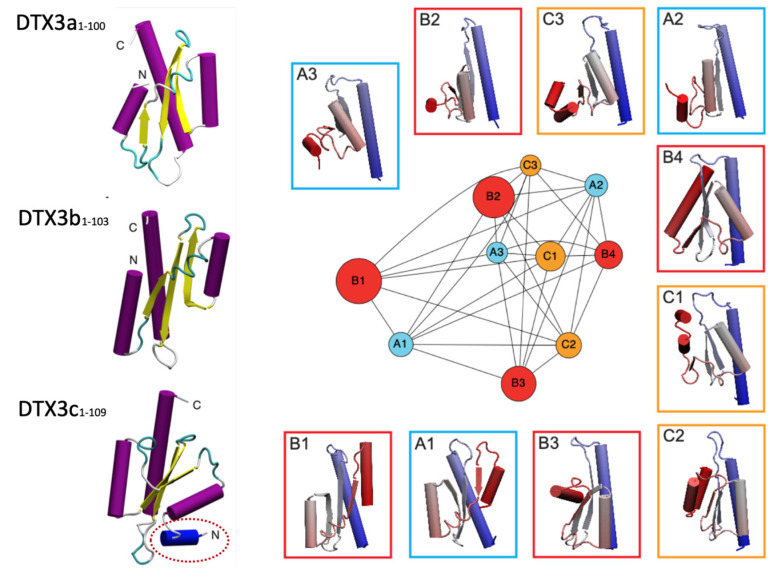
DTX3 isoforms N-terminal domain (N-terminal 100aa) predicted tridimensional structure. The structures projections shown on the left were selected to display the different N-terminal end orientation of DTX3c (in the red dotted circle) compared to isoforms a and b. The diagram on the right displays different projections of the DTX3 isoform Nt-100 structures shown on the left corresponding to the computational nodes (in the center) calculated out of 40,000 potential conformations (adapted from Scalia et al. 2023 [8]). The functional implications of DTX3c unique N-terminal spatial configuration are discussed in the text.

DTX1 bears two localization signal motifs spanning, respectively, aa 161–172 in domain I and aa 379–390 in domain II [25] (Figure 3). We were able to reproduce this finding using a previously described bioinformatic approach [26]. This approach recognized aa 164–172 along with the previously described 379–390 sequence (underlined in Table 2). This is consistent with the finding that, despite its demonstrated presence in the cytoplasm of cultured cells under standard cultural conditions [17,20,27], DTX1 has been found predominantly localized in the cell nucleus of a number of cellular models [27]. The parallel finding that DTX1 interacts directly with co-activator p300 and competes with it towards transcriptional activation of a number of genes both in a NOTCH-dependent and independent modality, has led to regard DTX1 as a constitutional transcriptional regulator [27] along with its demonstrated UbE3L mono-ubiquitination [14] and signaling scaffolding activities [28]. 

#### 2.4.2. DTX2

DTX2 cellular distribution has been reported predominantly in the nucleus of mammalian cells under standard cultural conditions [17,27]. Nonetheless, we found no NLS/NES motifs using an available NLS/NES search tool [26], nor we found any published evidence or mention of any NLS/NES motif in DTX2 both in UniProt and NCBI databases. It is therefore feasible that DTX2 nuclear localization is associated to an indirect unknown shuttling mechanism. 

#### 2.4.3. DTX3 

Similarly to DTX2, we found no published evidence of NLS/NES motifs in DTX3. Accordingly, our bioinformatic search using the cited algorithm [26] did not provide results to this effect. Preliminary unpublished data conducted in a mesothelioma cell line by the reviewers displayed cytoplasmic distribution of DTX3 with negligible nuclear signal under standard cultural conditions. Nonetheless, these results on endogenous DTX3 cellular staining were generated on a cell line (MSTO211H) found to express exclusively the DTX3c isoform [8], raising the question of whether this cellular distribution is shared by the other two known isoforms (DTX3A and B). Given the apparent lack of NLS motifs in DTX3 (Table 2) along with its association with renown nuclear targets such as TP53 and XRCC5 (Table 3), it will be relevant to determine the mechanistic and contextual mechanism for DTX3 nuclear translocation. In this context, DTX3 nuclear translocation may indeed be driven by DTX3 ability to form heterodimers with other NLS-containing DTX proteins [29]. This would potentially allow the DTX-bearing NLS motifs (DTX1 and DTX4) to act as nuclear shuttling partners independently from other demonstrated non-DTX cellular binding nuclear proteins reviewed herein (Table 3 and Table 4).

#### 2.4.4. DTX3L

Although two NLS and one NES motifs have been reported within the long N-terminal domain of DTX3L in an earlier review article [30], we were not able to find any source for such NLS/NES motifs in the cited article. Furthermore, the sequence screening using the above mentioned bioinformatic approach [26] did not produce any result (Table 2). As for the observed DTX3L cellular localization, cumulatively available evidences [14,17,31] has shown a predominant cytoplasmic distribution with a relative nuclear increase upon specific stimuli, such as in response to viral infection/interferon [14,31]. Indeed, the ability of DTX3L to dimerize with DTX1, which bears two NLS motifs (Table 2), could justify *per se* the shuttling of DTX3L to the nucleus. However, the molecular mechanism allowing its nuclear translocation is currently unclear.

#### 2.4.5. DTX4 

To date no NLS/NES motif has been reported in the literature for DTX4. Interestingly, using the NLS prediction algorithm based on Markov model [26] we could find an NLS motif in DTX4 spanning positions 378–388 of its Uniprot/NCBI-indexed protein sequence (Table 2). This predicted NLS motif resides within the proline rich region (domain-II) just upstream to the H2-RING domain, as graphically represented in Figure 1. The biological relevance of this finding towards DTX4 cellular and cancer-related functions will require further experimental evidences. Of note is that the predicted NLS motif should be preserved in the WWE-domain partially deficient DTX4 isoform, suggesting a diversified range of nuclear targets and contextual functions for this DTX4 variant. As for the reported cellular localization of DTX4, the only available evidence as a result of exogenous overexpression in HEK293 supports its predominant cytoplasmic distribution under standard cultural conditions [Ahmed 2015]. These observations suggest that specific stimuli may underlie its nuclear translocation.

The predicted/verified presence of NLS/NES in DTX proteins along with the observed cellular localization of DTX family members is presented in Table 2 below.

**Table 2:** NLS/NES motifs in DTX proteins and observed cellular localization.

Name	Predicted/Verified NLS Motif	Probability	Observed Cellular Localization	References
DTX1	161-RTQRRRRRLRRR-172 379-RKTKKKHLKKSK-390	P [0~1]:0.815 P [0~1]:0.887	Cytoplasmic ≤ nuclear	Yamamoto 2001 [27] Ahmed 2020 [17] Nguyen et al., 2009 [26]
DTX2	No predicted motif(s)	n/a	Cytoplasmic ≤ nuclear	Yamamoto 2001 [27], Ahmed [17] Nguyen et al., 2009 [26]
DTX3	No predicted motif(s)	n/a	Cytoplasmic ≥ nuclear	Scalia at al personal observations Nguyen et al., 2009 [26]
DTX3L	2 NLS + 1 NES reported Despite no predicted motif(s) found in present analysis	n/d	Cytoplasmic > nuclear	Zhang et al., 2015 [31] Nguyen et al., 2009 [26] Wang et al., 2021 [30]
DTX4	378-KTTKKQAKKGK-388	P [0~1]:0.758	TBD	Nguyen et al., 2009 [26]

## 3. DTX Proteins Signaling in Mammalian and Cancer Cells: A Phosphorylation, ADP-Ribosylation and Ubiquitination Network

A growing number of studies support a biological scenario in which DTX proteins, through their heterodimers and specific degradation complexes, establish molecular switches at their regulated protein target sites controlled by finely-tuned and coordinated ubiquitination, (de)phosphorylation and ADP-ribosylation events. Indeed, the link between DTX proteins and NOTCH can also be considered part of a coordinated ubiquitin regulatory network. Specifically, dDTX effects on NOTCH signaling were counteracted by Su(Dx), another class of UbE3Ls. Su(dx) [32,33] in fact belongs to a Nedd4 class of HECT domain proteins which, when co-expressed with DTX in *Drosophila*, is able to revert DTX actions with a dominant and tissue contextual fashion [33]. Based on the overall sequence homology and domains conservation between *Drosophila* DTX and its human orthologs (DTX1, DTX2, DTX4), it is possible that the effects of the human paralogs may also be counterbalanced by a corresponding Su(dx) human orthologs such as ITCH/AIP4 [34,35]. The specific role of human DTX proteins in cancer-related context is discussed below with respect to their targets and upstream regulators. The discussed findings have been also conveyed in Table 3 and Table 4. 

### 3.1. DTX1

Cloning of human DTX1 [20] has confirmed the close resemblance of its gene sequence and structural organization to the *Drosophila* ortholog, along with its ability to regulate NOTCH-dependent transcription. Accordingly, DTX1 mediates ubiquitination and promotes degradation of a number of targets belonging to NOTCH-associated as well as NOTCH independent signals (summarized in Table 3). Furthermore, human DTX1 displays both positive and negative regulatory effects over NOTCH-signaling in a variety of developmental and cellular contexts [13,36]. As for DTX, human DTX1 has been found to bind NOTCH1 via its WWE-domain-containing N-terminal region and to interact with eIF3f [28]. This interaction with eIF3f has been shown to be critical for NOTCH1 apparent deubiquitination. Indeed, this step leads to an active form of monoubiquitinated NOTCH with DTX1 acting as a mono-ubiquitination and bridging factor [28]. This cytoplasmic DTX1-mediated process has been suggested to play a key role to allow NOTCH nuclear translocation. It also assigns a key new role to the translation initiator factor eIF3f as a constitutive signaling component of the DTX1-NOTCH axis [28]. As discussed earlier, the SH3 motif located on Domain II (Figure 1), has been shown to interact with the signaling adaptor Grb2 [20] supporting potential regulation by a number of upstream RTK-generated signals [37]. DTX1 has also been described to form heterodimers with DTX3L [14]. Interestingly, DTX1/DTX3L heterodimerization enhances DTX1 ubiquitin ligase activity in vitro. Among DTX1-regulated cellular mechanisms involving its UbE3L activity are the PI3K/AKT and MEKK/MAPK pathways along with a number of transcription factors such as HES1, HIF1a, NFAT and c-FLIP (see Table 3). DTX1 is regulated itself by ubiquitination and UPS-mediated degradation via ITCH/AIP4, a human paralog of *Drosophila* Su(Dx) [34]. In an osteosarcoma cell model (OS187), DTX1 downregulated NOTCH1 and Hes1, and blocked invasiveness [38]. Conversely, DTX1 overexpression has been shown to confer oncogenic activity in glioblastoma cell lines (U373, LN18) and correlated with ERK activation [39]. In addition, its expression was inversely correlated with survival in both Glioblastoma and Breast cancer patients consistent with its cancer-promoting effect [39]. These findings support an oncogenic role for DTX1 in cancer. Most likely, DTX1 expression *per se* may not be sufficient to either drive or suppress cancer progression, and additional studies are needed to clarify its contextual role in cancer.

### 3.2. DTX2

The involvement of DTX2 in cancer has been initially suggested from the isolation of the cancer-promoting fusion gene product RUNX1-DTX2, as a result of the t (7;21) (q11.2;q22) chromosomal translocation in a set of acute myeloid leukemia patients’ cells [40]. DTX2 has been described to bind NOTCH1 and inhibit Wnt signaling [41]. A distinctive feature associated to the function of DTX2 has recently come from the demonstration of its ability to recruit and ubiquitinate poly-ADP-ribosylated (PAR) proteins through its domain-III, which encompasses both its H2-RING and the extended C-Terminal region [17,18]. Interestingly, such ability to recruit PAR proteins for other RING proteins (RFN146) has also been shown to require WWE motifs [42], which potentially expands WWE-domain function in DTX proteins beyond the NOTCH ankyrin repeats interaction. Since this PAR-binding sequences are shared with all other DTX family members, it has been suggested that DTX2 prevalent nuclear localization may act as a major contextual determinant for DTX2 preferred nuclear targets compared to the other DTX members, which are localized in the cytoplasm (Table 1) [17]. Despite the DTX2 general role in cancer is still unclear, some evidences support a cancer-driving effect. Specifically, an early study in colon cancer positively associated DTX2 expression with post-surgical relapse and poor outcome [43]. By the same token, another study in colon cancer cells (SW620, LoVo) [44] showed DTX2 to promote migration/invasion, increase NOTCH2 levels and trigger AKT activation. Poor survival correlation with DTX2 was observed also in Glioblastoma multiforme along with the demonstration that DTX2 silencing in glioma cell lines (DBTRG, U251) inhibited both their growth and migration abilities [45]. The same study found DTX2 transcriptional activation to depend on BRD4 binding to the DTX2 promoter. Another general mechanism by which DTX2 may promote cancer progression has been recently suggested by an inducible CRISP/Cas9 screen study disclosing DTX2 promoting role for hTERT transcription and tumorigenesis [46]. This effect of DTX2 is exerted through ubiquitination of NFIC leading to formation of a DTX2-NFIC complex and to the cooperative stabilization of NFIC binding to hTERT promoter. Additional studies are needed to clarify the biological weight of DTX2 on telomeres maintenance in a variety of cancer cell types. In hepatocarcinoma cellular models (HepG2 and Huh7) DTX2 physically interacted with DNA-binding protein RFX6 [47]. Furthermore, RFX6-DTX2 binding affected DTX2 own gene transcription at the promoter level, and increased DTX2-mediated NOTCH1 gene expression [47] (Table 4). As mentioned earlier, DTX2 displays two major isoforms which include its full length variant (622 aa, 67.2 kDa) and a shorter variant (575 aa, 62.3 kDa) differing in the presence of a peptidic stretch in Domain-II containing a proline-rich motif (missing in DTX2B) (Figure 1). Although studies addressing the biological differences between the two known DTX2 isoforms are missing, further investigation will eventually clarify each variant’s role and eventual involvement in modulating cancer behavior.

### 3.3. DTX3

DTX3, as a homodimer, is expressed in three N-terminal isoform variants (DTX3a, DTX3b, and DTX3c). The three isoforms are discussed individually below.

DTX3a (NM_178502) differs from the other isoforms in which it displays 7 distinctive N-terminal aa which are replaced in DTX3b by 10 different ones. The latter (DTX3c) instead originates as a result of an exon-retention event with the DTX3b N-terminal 10 aa followed by the 7 DTX3a N-terminal aa and a K->R single residue substitution at position 16 [8,48]. Interestingly, at the time of the present review, with a few exceptions, DTX3 has been found associated with tumor suppressing effects in human cancer. One of these exceptions is offered by a study correlating increased copy number of DTX3 in a cohort of breast cancer patients where DTX3 was associated with a cancer-driving effect [49] along with poor outcome. Similarly, another study focusing on Low Grade Glioma, associated DTX3 expression along with CDK4 and AVIL with a sub-group of patients with poor outcome [50]. On the opposite side, a study in esophageal carcinoma cells (KYSE150, TE-1, Eca-109) with exogenous expression of DTX3 exerted tumor suppressing functions [51] and found DTX3 to interact, ubiquitinate and partially colocalize with NOTCH2. This finding raises the question on which domain in DTX3 mediates the interaction with NOTCH2, since the WWE domain responsible for the physical binding with NOTCH2 ankyrin motif, are missing in DTX3 (and in DTX3L) (Figure 1). Another possibility to explain the results of this DTX3-NOTCH axis in esophageal cancer cells, supported by other studies on DTX3 heterodimeric [14] and heterogenous complexes formation [8] is that DTX3 may require an additional component in order to bind and regulate NOTCH in vivo. In fact, the two-hybrid approach used in the cited study does not exclude such possibility as compared to a cell-free reconstitution pull-down system. In triple negative breast cancer cells (TBNC), the RNA-binding protein DCAF13, a TNBC invasion-promoting factor, binds DTX3 mRNA 3′UTR and promotes its degradation causing NOTCH4 pathway activation [52]. DTX3 displayed tumor-suppressing effects also in colorectal cancer cells via regulation of E2F1 and its cell cycle-target genes [53]. DTX3 along with DTX4 have been found regulated by E2F transcription factors [54] (Table 4). However, in the cited studies, no indication of the involved DTX3 isoform was specified, leaving open the possibility of an isoform-specific role for the observed effects; a partial explanation of DTX3 isoforms role in cancer can be inferred and/or directly suggested by a few recent studies which have addressed (unknowingly) this issue. In particular, a recent study describing the exogenous expression of DTX3a (confirmed by the sequence of the cited expression construct) in ovarian cancer cells with different TP53 status, found DTX3a to bear tumor suppressing activities in wild type bearing TP53 cells, while promoting cancer effects in TP53 mutant ovarian cancer cells [55]. Interestingly, the study shows that (a) overexpression of DTX3a increased TP53mt levels in ES2 cells, and (b) DTX3 expressed in TP53mt-bearing ovarian cancer cells (OVCA420) was able to stabilize TP53mt levels. These findings further support the contextual cancer-promoting effect of DTX3a depending on the TP53 mutational status. All these effects were associated with direct binding of DTX3a with mtTP53, increase of its ubiquitination levels, and partial displacement of MDM2-mtTP53 binding.

DTX3b. As stated earlier, this isoform differs for the first N-terminal 10 amino acids which sequence is replaced by 7 distinctive aa in DTX3a (MPILSSSGSK->MSFVLSR). No specific experimental data are yet available regarding differential effects of DTX3b under both normal and cancer contexts. However, as shown by Scalia et al. [8] and in Figure 2 herein, a tridimensional modeling study out of 40,000 possible structural conformations, suggest a similar N-Terminal folding for both DTX3b and DTX3a isoforms compatible with similar biological effects. Nonetheless, such hypothesis still lacks specific experimental evidences. 

DTX3c. A new scenario has been recently added by the discovery of DTX3c (GenBank AXL48294.2) [48]). In particular, this new DTX3 isoform associates with a marked EphB4 depletion upon block of the autocrine IGF-II tyrosine phosphorylation signal rescuing EphB4 from its rapid degradation via its C-terminal *pi*Degron. Neutralization of cancer secreted IGF-II in these cells (a) blocked EphB4 Y987 phosphorylation, (b) increased EphB4 ubiquitination, and (c) associated with rapid and marked EphB4 protein clearance [7,8]. Such EphB4 degradation pattern upon autocrine IGF-II signal block was not so effective in HelaS3 cells, which express DTX3a. Interestingly, DTX3c recruitment by EphB4 C-*pi*Degron upon IGF-II deprivation required the intact unfoldase activity of the chaperon Cdc48/p97 (also known as VCP). The DTX3c behavior in this study was clearly opposed to the cancer-promoting effects linked to IGF-II- /EphB4 overexpression described in the literature [56,57] support a tumor-suppressing role for DTX3 similar to other studies reviewed herein in cancer cellular models. A tridimensional rendering of DTX3 isoforms N-terminal region displaying the unique folding of DTX3c differentiating it from the previously known DTX3 isoforms is shown in Figure 3 below.

### 3.4. DTX3L

DTX3L has been initially found to form heterodimers with DTX1 [14], as mentioned previously and conveyed in Table 3. It has been found over-expressed and to bind Parp9 (BAL1) in Diffuse Large B-Cell Lymphoma (DCBCL) [58]. DTX3L-Parp9 interaction has been found necessary for mono-ADP-ribosylation of ubiquitin which inhibits DTX3L UbE3L function in DNA repair. On the opposite side, NAD+-dependent binding of Parp9 to poly-ADP-ribose enhanced DTX3L UbE3L activity [59]. The DTX3L-mediated DNA repair effect has also been associated to its ability to exert mono-ubiquitination of histone H4 [60]. In PC3, DU145 and NNCAP prostate cancer cells, DTX3L promoted proliferation and chemo-resistance through its binding to ADP-ribosyl transferases Parp9 and Parp14 and by inhibiting IRF1 [61]. DTX3L is highly expressed in human melanoma tissues and cell lines (MNT1, G361, A375P, A375M, SK-Mel28) where it stimulated metastatic effects associated to activation of the FAK-PI3K-AKT axis [62]. In cervical carcinoma cell lines (HeLa, SiHa) DTX3L knockdown inhibited the PI3K-AKT-mTOR axis, suppressed proliferation, invasion, xenograft tumorigenesis and synergized with Cisplatin towards drug-induced apoptosis [63]. Among other cellular processes regulated by DTX3L is the regulation of CXCR4 endosomal sorting which limits the rate of its ubiquitination and degradation by other E3 ligases [64]. The implication of such effect in cancer biology is still to be determined. DTX3L has also been found up-regulated by METTL3, an RNA N6-methyladenosine (m^6^A) transferase [65] In particular, downregulation of the METTL3-DTX1/DTX3L dimer axis, upregulates NOTCH signaling, impairs angiogenesis in vitro, and correlates with cerebral arteriovenous malformations [65]. These DTX3L effects via the described signaling partners have been summarized in Table 3 and Table 4.

### 3.5. DTX4

DTX4 has been found associated with a number of solid cancers including colorectal cancer [66], hepatocellular carcinoma [54], and melanoma [67], via Notch-dependent and independent mechanisms. However, to date, the most direct mechanistic evidence of DTX4 involvement in cancer relates to the regulation of the oncogenic factor TBK1 as a direct degradation target of DTX4 UbE3L activity. TBK1 is an IRF3 activator which is also activated by RAS mutants-generated signals (reviewed by Runde et al. [68]). Specifically, DTX4 has been found to target TBK1 for degradation in response to NLRP4, as part of the IFN-mediated response [69]. TBK1 regulation by DTX4 has been further confirmed via demonstration that DYRK2 promotes TBK1 degradation via serine 527 phosphorylation which is essential for the recruitment of the NLRP4/DTX4 degradation complex [70], and by the observation that TBK1-degradation by TRAF3IP3 requires DTX4-mediated K48 ubiquitination of TBK1 [71]. As such, TBK1 constitutes a valuable DTX4-regulated oncogenic protein. DTX4 is also involved in adipogenesis since it promotes lipid incorporation and pre-adipocytes clonal expansion via C/EBPα; and PPARγ [72]. Interestingly, in this study DTX4 silencing increased the RNA transcripts levels of WNT6, WNT10 and β-Catenin in preadipocites (3T3-L1). DTX4 has also been found negatively regulated by cMYB [73] and Let7a [74] at the transcriptional and post-transcriptional level, respectively (Table 4). Given the individual involvement of WNT family members cMYB and Let7a in the tumorigenic process, these findings set the basis for further studies focusing on DTX4 and its specific role in cancer regulation.

**Table 3:** Cellular targets regulated by human DTX proteins.

DTX Protein	Regulated Target	Reported Effect(s)	Physio-Pathological Context	DTX Isoform Involved	Reference(s)
DTX1	NOTCH1	DTX1 downregulate NOTCH1 by ubiquitination of ICN	OS187 (osteosarcoma)	n/a	Zhang et al., 2010 [38]
	MEKK1	DTX1-mediated degradation	T-cell activation	n/a	Liu et al., 2005 [75]
	eIF3f	Docks DTX1 to NOTCH1 and acts as a DUB for NOTCH1	U2OS	n/a	Moretti et al., 2010 [28]
	HES1	DTX1 reciprocal inhibition	Osteosarcoma	n/a	Zhang et al., 2010 [38]
	PI3K/MAPK pathways	Cancer-promoting effect via mitogenic signaling activation	Glioblastoma	n/a	Huber et al., 2013 [39]
	PKCθ	DTX1-mediated degradation	T-cells	n/a	Hsu et al., 2014 [76]
	HIF1α	DTX1-induced, VHL-mediated HIF1α degradation	T-cell activation, immunotolerance	n/a	Hsiao et al., 2015 [77]
	PI5P4Kγ	Is a DTX1 target and bears opposite effects on NOTCH	Hela, U2OS cellular models	n/a	Zheng et al., 2015 [78]
	c-FLIP	DTX1-mediated degradation	Gastric cancer	n/a	Hsu et al., 2018 [79]
	NFAT	Increased DTX1 expression enhances NFAT transcription via activation of p38MAPK	HIV-infected T-Cells	n/a	Che et al., 2012 [80]
DTX2	NOTCH1	Notch1 antagonized β-catenin activity through its interaction with Deltex-2	HCT116 (Colorectal ca)	N.S.	Acar et al., 2021 [41]
	Parp1/2	DTX2 interacted and Ubiquitinylated Parp1/2 in the nucleus	HEK293 Hela (Cervical ca)	N.S.	Ahmed SF et al., 2020 [17]
	XRCC5-XRCC6	DTX2 interacted and Ubiquitinylated XRCC5/6 in the nucleus		N.S.	
	SPT16	DTX2 interacted and Ubiquitinylated SPT16 in the nucleus		N.S.	
	MYOD	DTX2-induced repression of MYOD via Jmjd1c	Myogenic differentiation	N.S.	Luo et al., 2017 [81]
DTX3	NOTCH2	TX3-induced ubiquitination	Esophageal Ca	N.S.	Ding et al., 2020 [51]
	XRCC5	DTX3-mediated EMT inhibition via XRCC5 ubiquitination	Thyroid Ca	DTX3a *	Wang et al., 2021 [82]
	TP53	DTX3 expression increases TP53 stability and levels	Ovarian Ca	DTX3a *	(Wang et al., 2022) [55]
	EphB4	IGF-II-inhibited, DTX3-mediated EphB4 degradation	Malignant Mesothelioma	DTX3c	Scalia et al., 2023 [8]
DTX3L	NOTCH1	Downregulated NOTCH1 acting as a dimer with DTX1	DLCBL cell lines	FL-DTX3L (isoform 1)	Takeyama et al., 2003 [14]
	Parp9	Forms heterodimer with Parp9 to (a) increases DNA repair, and (b) control viral infection	DNA repair in Prostate Ca/ Interferon signaling	FL-DTX3L (isoform 1)	Yang et al., 2017 [59] Zhang et al., 2015 [31]
	IRF1	Inhibits IRF1 tumor suppressor via complex formation with ARTD9 and STAT1	Prostate Ca	FL-DTX3L (isoform 1)	Bachmann et al., 2014 [61]
	TP53	As heterodimer with Parp9 it targets TP53 for degradation at DNA damaged sites	U2OS engineered Cellular model	FL-DTX3L (isoform 1)	Yan et al., 2023 [83]
DTX4	NOTCH1	DTX4 ubiquitinates NOTCH1 prior to ADAM10 processing	Selected cellular models	N.S.	Chastagner et al., 2017 [84]
	TBK1	NLRP4-induced, DTX4-mediated TBK1 degradation	Interferon signal regulation	N.S.	Cui et al., 2012 [69]; An et al., 2015 [70]; Deng et al., 2020 [71]
	C/EBPα PPARγ	DTX4 silencing downregulates C/EBPα and PPARγ	adipogenesis	N.S.	Wang et al., 2017 [72]

* DTX3 isoform determined on the basis of the sequence of the expression construct used for the cited study.

**Table 4:** Transcriptional and/or post-transcriptional regulators of DTX family members.

DTX Protein	Regulator Name (Type)	Regulatory Effect	Model/Pathological Context	Reference(s)
DTX1	ITCH/AIP4 (E3-ub-ligase)	DTX1 ubiquitin-mediated degradation	Cellular model (HEK293)	Chastagner et al., 2006 [34]
	NFAT	NFAT increases DTX1 transcription	T-cells	Hsiao 2009 [85]
DTX2	BRD4	Transcriptional regulation	Glioblastoma	(Wu et al., 2022) [45]
	RFX6	Transcriptional regulation	Hepatocellular Ca	Song et al., 2021 [47]
DTX3	DCAF13 (RNA binding protein)	DTX3 mRNA degradation	TN Breast ca promotion	Liu et al., 2020 [11]
	E2F1/E2F3 (transcription factors)	DTX3 Inhibits E2F1-mediated transcription	Colorectal Ca Hepatocellular Ca	Xu et al., 2022 [53] Viatour et al., 2011 [54]
DTX3L	METTL3	DTX3L mRNA stabilization	Human endothelial cells (HUVEC)	Wang et al., 2020 [65]
DTX4	Let7a (miRNA)	Negatively regulates DTX4 mRNA	Ureters-pelvic obstruction	Papadopoulos et al., 2017 [74]
	E2F1/E2F3 (transcription factors)	Transcriptional regulation	Hepatocellular Ca	Viatour et al., 2011 [54]
	cMYB (Transcription Factor)	Negative Transcriptional regulation	Liver Kupffer cells	Wu et al., 2023 [73]

## 4. Conclusions and Perspectives

Our understanding of the full biological role of DTX proteins is just at its early stage. More clarity is expected to come from additional areas of investigations focusing on the identification of novel DTX-regulated proteins, along with the description of the targeted degrons/phospho-degrons and the underlying regulated signaling pathways. No less important will be the clarification of the functional post-translational-modification-network underlying DTX protein signals and molecular functions. The recently published literature indeed already offers a number of DTX-regulated pathways and cellular biochemical processes greater than that postulated just a few years ago. Accordingly, all DTX members have been found to play a role in cancer, where they exert either cancer-driving or tumor-suppressing effects. Among them, DTX3 seems to favor a predominant tumor-suppressing role in the studied cultured cancer cell models. However, specific studies should address this question for all DTX family members in the light of their demonstrated ability to elicit both DTX hetero-dimer-specific and non-DTX complex-associated functions. Finally, the recent identification of a new DTX3 isoform (DTX3c), and this DTX3 isoform-specific association with the regulation of EphB4 levels in cancer [8], discloses an additional chapter in the yet under-exploited role of DTX isoforms in cancer biology. Such cumulative understanding will be of great help toward envisioning and designing modulatory strategies toward personalized treatments in those cancer types where DTX proteins are found expressed.

## Figures and Tables

**Table 1:** Human DTX genes and relative products features ^1^.

Name	Alternative Names	Gene Locus	UniProt Reference ID	Full Length Variant MW	Dimer Formation
DTX1	--	12q24.13	Q86Y01	67,368 Da	DTX1/ DTX3L
DTX2	KIAA1528 RNF58	7q11.23	Q86UW9	67,246 Da(A) 62,344 Da(B)	Homodimer
DTX3	RNF154	12q13.3	Q8N9I9	37,988 Da(a) 38,155 Da(b) 38,847 Da(c)	Homodimer
DTX3L	BBAP	3q21.1	Q8TDB6	83,554 Da	DTX1/ DTX3L
DTX4	KIAA0937 RNF155	11q12.1	Q9Y2E6	67,258 Da	Homodimer

^1^ As from UniProtKB unless otherwise specified.

## Data Availability

Not applicable.
